# Comparative study of the cytotoxicity, apoptotic, and epigenetic effects of Boswellic acid derivatives on breast cancer

**DOI:** 10.1038/s41598-022-24229-y

**Published:** 2022-11-21

**Authors:** Fatemeh Jamshidi-adegani, Shokoofeh Ghaemi, Sulaiman Al-Hashmi, Saeid Vakilian, Juhaina Al-kindi, Najeeb Ur Rehman, Khurshid Alam, Khamis Al-Riyami, Rene Csuk, Ehsan Arefian, Ahmed Al-Harrasi

**Affiliations:** 1 Laboratory for Stem Cell and Regenerative Medicine, Natural and Medical Sciences Research Center, University of Nizwa, P. O. Box: 33, 616 Nizwa, Oman; 2 Department of Microbiology, School of Biology, College of Science, University of Tehran, Tehran, Iran; 3 Natural Products Laboratory, Natural and Medical Sciences Research Center, University of Nizwa, P. O. Box: 33, 616 Nizwa, Oman; 4 Department of Mechanical and Industrial Engineering, Sultan Qaboos University, 123 Muscat, Oman; 5 Organic Chemistry, Martin-Luther-University Halle-Wittenberg, Kurt-Mothes-Str. 2, 06120 Halle (Saale), Germany

**Keywords:** Breast cancer, Toxicology

## Abstract

This study aimed to compare the effect of Boswellic acid derivatives on the viability, apoptosis, and epigenomic profiling of breast cancer. According to the viability assays, 3-*O*-acetyl-11-keto-β-Boswellic acid (AKBA) showed more toxicity against MDA-MB-231 cells when compared with the 3-*O*-acetyl-β-Boswellic acid (ABA). In contrast, ABA revealed less toxicity against MCF-10A. Cell cycle and apoptosis assays determined the maximum apoptotic effect of AKBA on MCF-7, and MDA-MB-231 cells. Interestingly, β-Boswellic acid (BA) and ABA did not promote the apoptosis in MCF-10A cells. Transwell migration assay indicated the greatest normalized inhibition (around 160%) in the migration of MDA-MB-231 cells induced by AKBA. The expression of *P53*, *BAX*, and *BCL2* genes in cancerous cell lines has affirmed that both AKBA and ABA could induce the maximal apoptosis. Western-blot investigation demonstrated that the maximum over-expression of *P53* protein (1.96 times) was caused by AKBA in MDA-MB-231 cells, followed by ABA in MCF-7 cells. The *BCL2* protein expression was in agreement with the previously reported results. The global DNA methylation in both cancerous cells was reduced by ABA. These results suggest that ABA represented more epigenetic modulatory effect while AKBA shows more cytotoxic and apoptotic effect against breast cancer cell lines.

## Introduction

Breast cancer is considered one of the most lethal cancer among women due to its high rate of incidence and potential of metastasis^1^. It is obvious that the inception of mammary carcinoma is caused by the accretion of mutated genes which alter their functions^2^. These limited cellular functions can be seen as different criteria, such as inflammation, cell cycle control, detoxification, apoptosis, and cell migration. In fact, these mutations can lead either to the inactivation of tumor suppressor genes or activation of proto-oncogenes, which play an important role in transforming cells to the malignant state^2^. Recently it has been indicated that epigenetic, as a term which was first proposed by Waddington, plays a major role in tumor development as significant as genetic^3^. Tumor suppressor gene silencing induced by hypermethylation of CpG islands of genes, and global genomic DNA hypomethylation are considered as very important molecular pathways lead by DNA methylation^4^.

The efficacy of cancer chemotherapy is extremely restricted by the cellular resistance to multiple drugs, which are structurally and mechanistically unrelated^5^. The multidrug resistance (MDR) occurs principally because of ATP-binding cassette (ABC) transporter's overexpression in cancer cells leading to extruding medicines outside of cells^5^. Synthetic chemo-sensitizers have been developed to reverse the MDR by blocking ABC transporters, while none of them have been employed in clinic^6^. Natural chemo-sensitizers with low systemic toxicity can be applied either independently or as an adjuvant to promote the therapeutic effectiveness against MDR. This would take effect by boosting drug efficacy and minimizing the toxic dose level^7^. Late studies have supported the capability of phytochemicals including alkaloids, polyphenols, and flavonoids, to sensitize the triple-negative breast cancer cell line to revert MDR^7,8^.

The *Boswellia sacra* resin has been traditionally applied as a folk medicine to treat various diseases, including inflammatory and cancer disorders^9^. The underlying mechanism of anti-inflammatory activities is mostly related to pentacyclic triterpenic acids, namely boswellic acids which have been recognized as active ingredients of frankincense^10^. While the chemical structure of boswelic acids is similar to that of steroids^11^, they have indicated quite different properties from nonsteroidal anti-inflammatory drugs by the inhibition of 5-lipoxygenase^12^. Based on the previous in vitro and in vivo studies, the boswellic acid derivatives are well-recognized as active components responsible for the anti-cancer activities of frankincense^13^. Furthermore, they are reported to possess cancer chemo-preventive activities^14^.

β-Boswellic acid (BA), 3-*O*-acetyl-β-Boswellic acid (ABA), and 3-*O*-acetyl-11-keto-β-Boswellic acid (AKBA) are the major derivatives of Boswellic acids^15,16^*.* It has been proven that BA could induce apoptosis in colon cancer cells through a caspase-8-dependent pathway^17^. ABA revealed a more influential impact on the apoptosis of glioblastoma cells in comparison with the BA and AKBA^18^. Moreover, the whole-transcriptomic study of ABA indicated that it can induce apoptosis and inhibit epithelial-mesenchymal transition, cancer cell viability, proliferation, and metastasis by interference in the different molecular pathways^18^. ABA in particular showed a more influential anti-cancer impact against glioblastoma cells when compared with BA and AKBA^19^. As reported, ABA didn't indicate toxicity against normal cells, while it caused DNA fragmentation in melanoma cells^20^. Among boswellic acid derivatives, AKBA has been reported to have the strongest anti-cancer properties. AKBA could decrease chemotherapy resistance in human cancers, for example, AKBA decreased the malignancy of Taxol resistant human ovarian cancer by inhibiting multidrug resistance protein function and also, suppressed Docetaxel-resistant prostate cancer cells in vitro and in vivo by blocking Akt and Stat3 signaling^21,22^. We have recently investigated the apoptotic effects of AKBA against human prostate and breast cancer cells^23^.

Despite the promising anti-cancer activities, the low bioavailability of the boswellic acid derivatives, specifically AKBA reduces their pharmacological effectiveness due to their inadequate cellular and tissue uptake. To promote the pharmacodynamics properties of these compounds, several approaches have been employed. The administration of 11-keto-β-Boswellic acid (KBA) following a standardized meal strengthened the bioavailability of KBA^24^. The bioavailability of boswellic acid derivatives can be enhanced by the co-administration with an anionic drug^25^. Drug delivery system such as micelles, emulsions, lipid nanoparticles, lipid nano-carriers, liposomes and synthetic polymeric nanoparticles have been applied to improve pharmacodynamics effect of these compounds^26–28^. As reported by Riva et al., formulation of boswellic acids with lecithin could improve the tissue absorption and diffusion, resulting in the enhanced bioavailability^29^.

Based on recent studies, these compounds possessed a promising potential to be used as anticancer agent, while still their mechanistic effects on non-tumorigenic non-transformed breast epithelial (MCF-10A) and cancerous (MCF-7, and MDA-MB-231) cells are unclear. Therefore, in this comparative study, the impact of these bioactive compounds (BA, ABA, and AKBA) on MCF-10A cells and breast cancer cells was evaluated by the toxicity, cell cycle, cell migration, apoptosis, epigenetic, gene and protein studies.

## Results

### Cytotoxicity assay

To investigate the cytotoxicity effect of BA, ABA, and AKBA on MCF-7, MDA-MB-231, and MCF-10A cells, the viability of treated and non-treated cells was assessed using an MTT assay. Based on MTT results (the data not shown), an optimal concentration of drugs was selected to have the least effect on MCF-10A cells as well as the maximal inhibition effect on the proliferation of cancer cells. So, from the tested concentrations, we selected 65 μM concentrations for the next steps.

**Figure 1:**
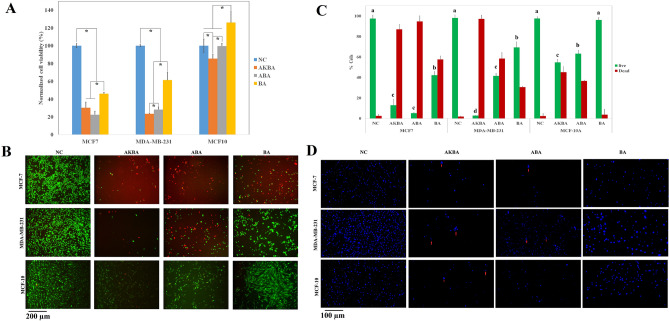
(**A**) the normalized viability of MCF-7, MDA-MB-231, and MCF-10A cells obtained by the MTT assay following 24 h treatment with AKBA, ABA, and BA in comparison with the negative control (NC). * indicated a significant difference (P < 0.05) (N value = 3). (**B**) Live and dead assay of MCF-7, MDA-MB-231, and MCF-10A after 24 h of treatment with AKBA, ABA, and BA in comparison with the negative control (NC). Green and Red are representing live and dead cells, respectively. (**C**) Quantitative analysis of live and dead staining of MCF-7, MDA-MB-231, and MCF-10A after 24 h of treatment with AKBA, ABA, and BA in comparison with the negative control (NC). a, b, c, and d indicate significant levels for the number of live cells (P < 0.05) (N value = 3). D. DAPI staining of MCF-7, MDA-MB-231, and MCF-10A after 24 h treatment with AKBA, ABA, and BA in comparison with the negative control (NC). Arrows represent apoptotic bodies.

As shown in Fig. 1A, the normalized viability of both cancer cells (MCF-7 and MDA-MB-231) was declined considerably when treated with BA, ABA or AKBA. There is no significant difference between the viability of MCF-7 cells treated with AKBA and ABA. On the other hand, MDA-MB-231 cells treated with AKBA have indicated less viability compared to the ABA. With regard to both cancerous cells, BA revealed less anti-proliferative effect in comparison with either ABA or AKBA.

In agreement with the MTT results, all three compounds (BA, ABA, and AKBA) revealed significant anti-cancer activities against MCF-7 cells (Fig. 1B). ABA and AKBA showed the maximal anti-cancer activity against MCF-7 cells with 87% and 95% of dead cells; however, the difference between them was not statistically significant (Fig. 1C). While approximately 97% of MDA-MB-231 cells were eradicated by AKBA treatment, just 58% of them were abolished using ABA. While ABA and BA have made no significant toxicity against MCF-10A cells, AKBA has led to a 15% decrement in the survival of MCF-10A cells (MCF-10A).

In parallel with MTT and live and dead assays results, DAPI staining (Fig. 1D) revealed an inhibition in the proliferation of MCF-7, MDA-MB-231, and MCF-10A cells induced by AKBA, and ABA. While in the NC group for both cancerous cell lines, the stained nuclei had round morphology, ABA- and AKBA-treated cancerous cell lines showed an altered nuclear morphology with apoptotic bodies (red arrows). In case of MCF-10A cells, BA-, and ABA-treated cells' nuclear revealed less apoptotic morphology compared to the AKBA.

### Cell cycle assay

**Figure 2:**
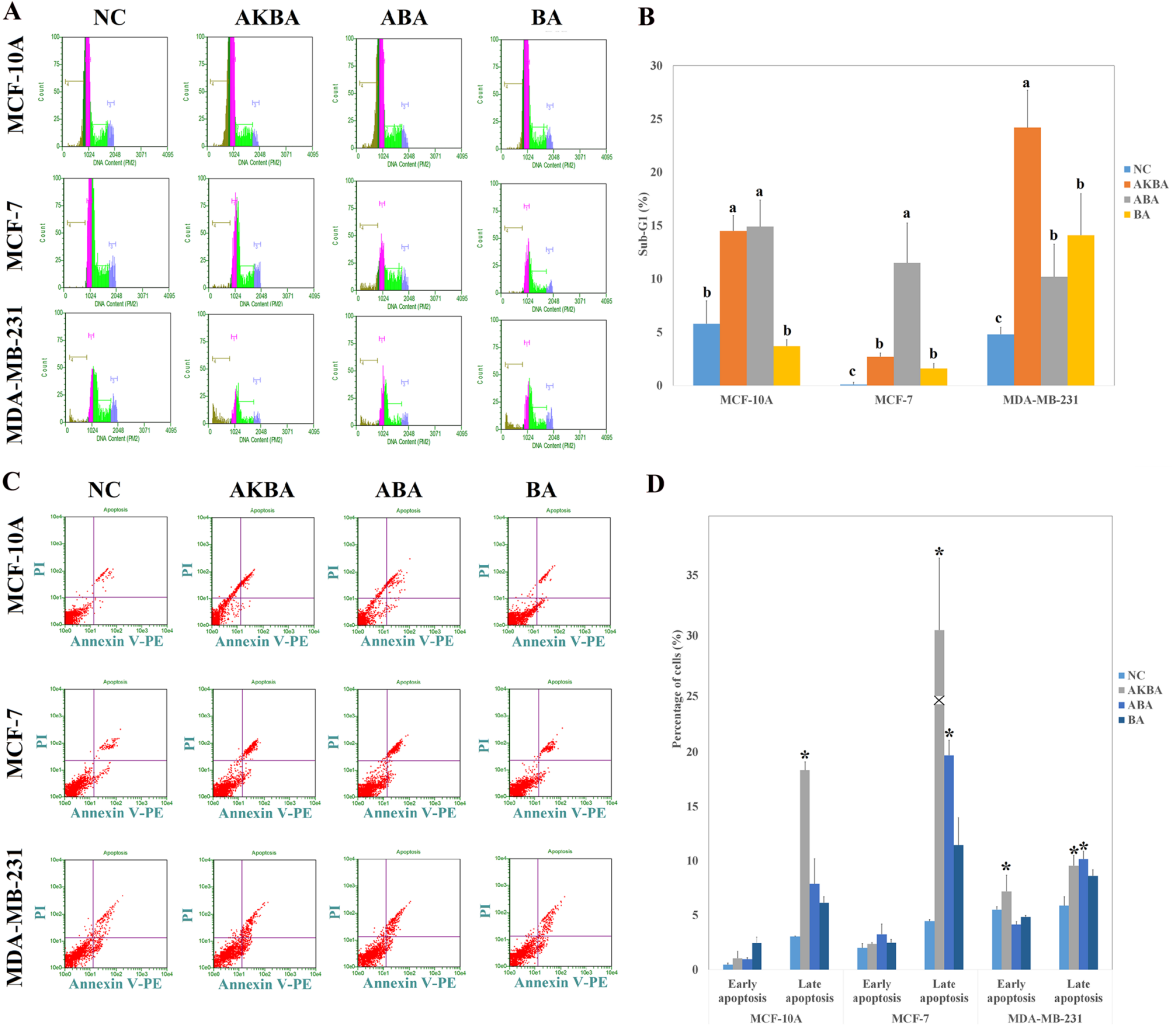
(**A**) Cell cycle assay of MCF-7, MDA-MB-231, and MCF-10A cells following 24 h treatment with AKBA, ABA, and BA in comparison with the negative control (NC). (**B**) The percentage of cells that are in the sub-G1 phase in MCF-7, MDA-MB-231, and MCF-10A after 24 h of treatment with AKBA, ABA, and BA in comparison with the negative control (NC). a, b, c, and d indicate significant levels for the percentage of cells in Sub-G1 phase (P < 0.05) (N value = 3). (**C**) Apoptosis assay of MCF-7, MDA-MB-231, and MCF-10A cells obtained by the Annexin V-FITC apoptosis detection kit following 24 h treatment with AKBA, ABA, and BA in comparison with the negative control (NC). (**D**) Percentage of apoptotic MCF-7, MDA-MB-231, and MCF-10A cells following 24 h treatment with AKBA, ABA, and BA in comparison with the negative control (NC). *indicates a significant difference in comparison with the NC (P < 0.05) (N value = 3).

As depicted in Fig. 2A,B, the effects of AKBA, ABA and BA on the proliferation of MCF-7, MDA-MB-231 and MCF-10A cells were further studied by cell cycle assay. In MCF-10A cells, the sub-G1 phase, which reveals that apoptotic cells were raised 3.6 and 4.8 folds upon treatment with AKBA and ABA compared with the negative control, respectively. On the other hand, there was no obvious effect seen by BA. Moreover, the sub-G1 phase in MCF-7 cells was increased 7.8 folds in AKBA treated cells in comparison with the negative control, whereas ABA and BA revealed a less effect on this cell line. Finally, the sub-G1 phase was enhanced 3.8 times in AKBA treated MDA-MB-231 cells compared with the negative control.

### Apoptosis assay

Using Annexin-V/propidium iodide apoptosis assay, the effect of BA, ABA, and AKBA on the early- and late-apoptosis of MCF-10A and cancerous cell lines were further investigated as shown in Fig. 2C,D. Whereas none of active compounds could affect the early-apoptosis of MCF-10A cells, AKBA significantly induced the late-apoptosis phase of MCF-10A cells (around 6 times more compared to the NC group). The represented apoptotic effect of AKBA against MCF-10A cells is in the agreement with the cell cycle assay result. None of the pure compounds had a significant effect on the early-apoptosis phase of MCF-7 cells. On the other hand, AKBA and ABA significantly strengthened the late apoptosis phase in MCF-7 cells 7 and 4.5 times compared with the control group, respectively. In the MDA-MB-231 cell line, both AKBA and ABA remarkably increased the late apoptosis phase (1.6 and 1.7 times compared to the control group, correspondingly), while only AKBA significantly induced the early-apoptosis phase.

### In vitro migration assay

**Figure 3:**
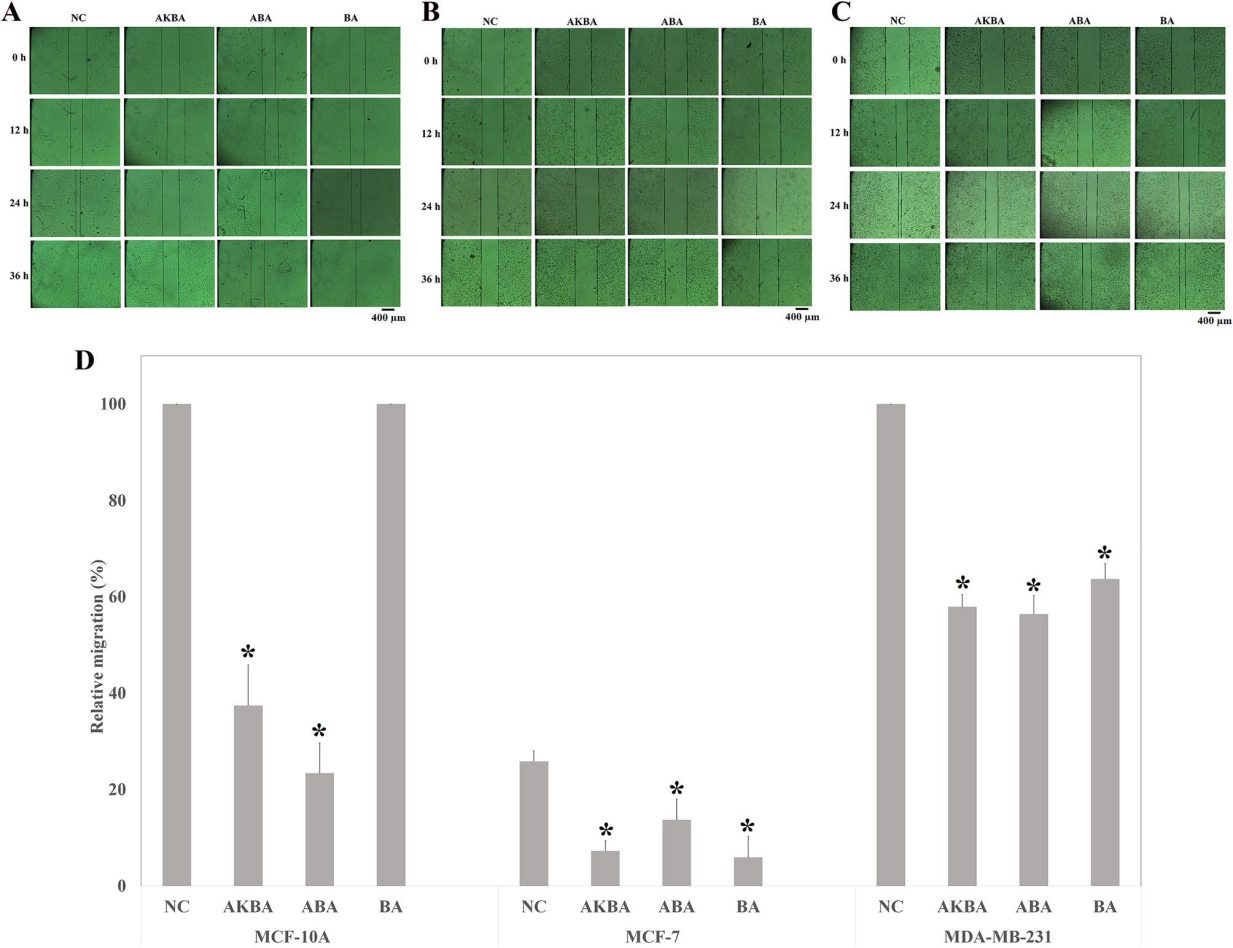
Scratch migration assay. The photos were taken 12, 24 and 36 h after treatment. (**A**) Scratch closure in MCF-10A cells treated with AKBA, ABA, and BA compared with the negative control (NC). (**B**) Scratch closure in the MCF-7 cells treated with AKBA, ABA, and BA compared to the NC. (**C**) Scratch closure in the MDA-MB-231 cell line treated with AKBA, ABA, and BA compared to the NC. (**D**) Quantitative analysis of treated- and non-treated- MCF-10A, MCF-7, and MDA-MB-231 cells' migration. *indicates a significant difference compared to the NC (P < 0.05) (N value = 3).

2D-scratch assay was employed to assess the effect of pure compounds on the migration of MCF-10A and cancerous cell lines (Fig. 3A–D). Although the migration of MCF-10A cells was significantly inhibited by AKBA and ABA, BA had no effect on MCF-10A cell migration (Fig. 3A). In the other words, MCF-10A cells migration was restricted by AKBA and ABA almost 66% and 84%, subsequently (Fig. 3D). 2D-scratch migration assay revealed that all compounds were capable to significantly hinder the migration process (p < 0.05), considerably in cancer cells (MCF-7 and MDA-MB-231) (Fig. 3B,C). As shown in Fig. 3D, 18.5%, 12%, and 20% of the migration of MCF-7 cells were inhibited by AKBA, ABA, and BA, subsequently. Additionally, the migration of MDA-MB-231 cells in 2D scratch assay was inhibited approximately 42%, 43.5% and 36% by AKBA, ABA, and BA compared to the control group, respectively.

**Figure 4:**
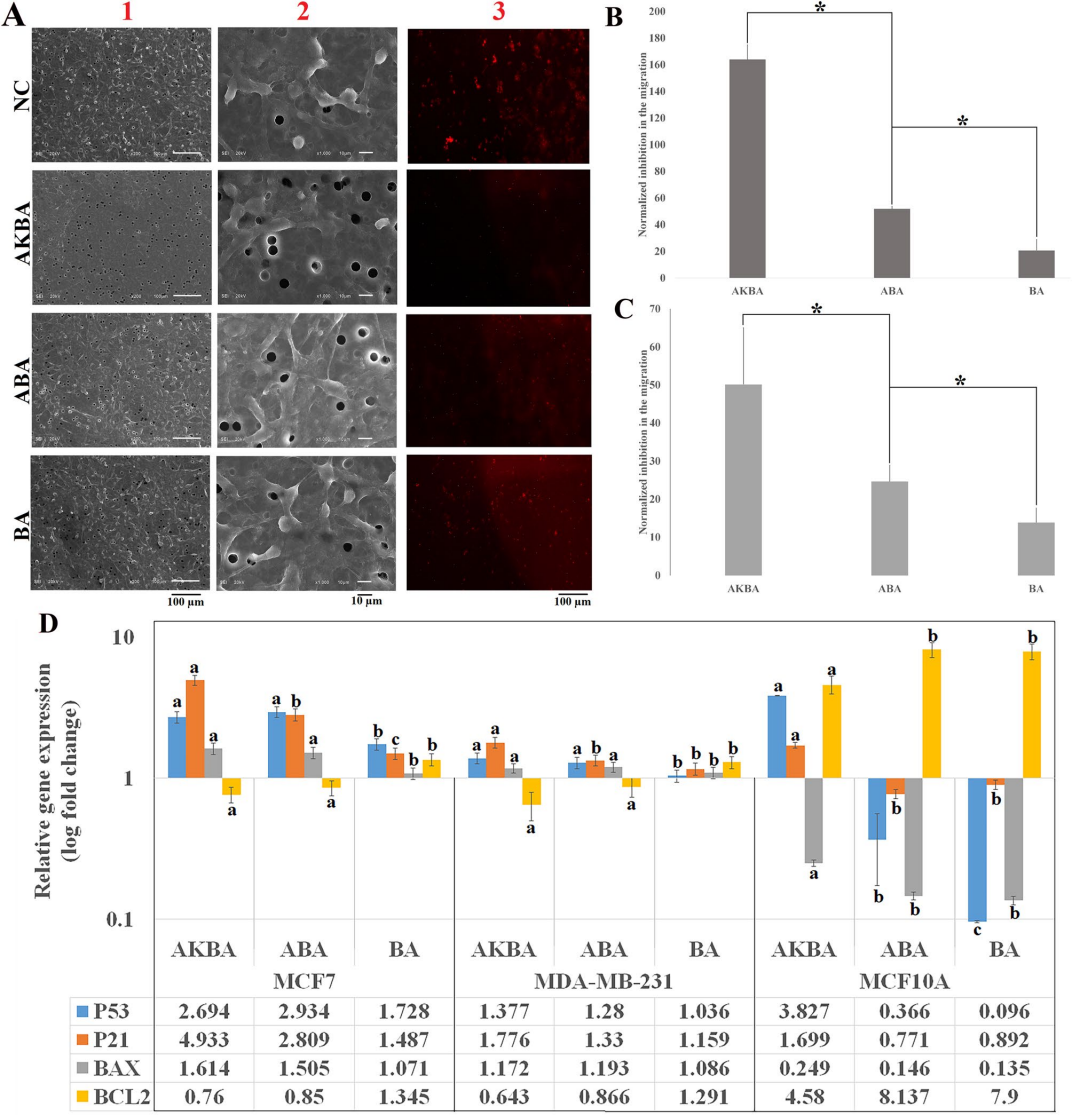
Transwell migration assay. (**A1, 2**) SEM photos from the migrated MDA-MB-231 cells from the lower side of the transwell membrane. These photos (200X, and 1000X) were taken 48 h after treatment the cells with AKBA, ABA, BA, and negative control (NC). (**A3**) Fluorescent imaging of red-labeled MDA-MB-231 cells that migrated to the lower side of the membrane. (**B**) Normalized inhibition in MDA-MB-231 cells migration obtained from quantitative analysis of SEM photos. *indicates a significant difference (P < 0.05) (N value = 3). (**C**) Normalized migration inhibition compared to the negative control, as obtained from the quantitative analysis of red-labeled cell migration (n = 3). (**D**) Relative gene expression of *P53*, *P21*, *BAX*, and *BCL2* genes in MCF-7, MDA-MB-231, and MCF-10A cells after 24 h treatment with AKBA, ABA, and BA in comparison with the negative control. a, b, c, and d revealed different significant levels in each gene (N value = 3).

As indicated in Fig. 4A, in parallel with the scratch assay, transwell migration assay results proved that AKBA, ABA, and BA could suppress the invasion of MDA-MB-231 cells. The quantitative analysis of transwell migration assay (Fig. 4B,C) has indicated that AKBA induced the maximal inhibition in the migration of MDA-MB-231 cells.

### Relative gene expression

As shown in Fig. 4D, the *P53* gene levels were over-expressed 2.7, and 2.9 times in AKBA- and ABA- treated MCF-7 cells compared with the NC, however, the difference was not statistically significant. On the other hand, *P21* gene was up-regulated 4.9 time in AKBA-treated MCF-7 cells in comparison with the NC, which was significantly higher than the ABA-treated MCF-7 cells (2.8 times). The *BAX* gene was induced remarkably in both ABA- and AKBA-treated MCF-7 cells at similar levels. The relative expression of *BCL2* as an anti-apoptotic gene was noticeably reduced in both ABA-, and AKBA- treated MCF-7 cells compared with the NC.

Similarly, in MDA-MB-231 cells, the relative gene expression of *P53*, *P21*, and *BAX* were significantly up-regulated in both ABA and AKBA groups. On the other hand, the *BCL2* gene was down-regulated 0.64, and 0.86 fold in ABA- and AKBA-treated MDA-MB-231 cells, respectively. The results suggested that both ABA and AKBA are able to induce apoptosis in MCF-7 and MDA-MB-231 cells through *P53*, *P21*, *BAX*, and *BCL2* gene levels regulation.

The levels of *P53* and *P21* were up-regulated only in AKBA-treated MCF-10A cells in comparison with the NC, suggesting the possible apoptotic effect of AKBA on MCF-10A cell line. Despite this, a drop in the relative gene expression of BAX gene was observed in AKBA-treated MCF-10A cells, albeit this reduction was significantly less than that of ABA- and BA-treated MCF-10A cells. *BCL2* as an anti-apoptosis gene was up-regulated in all treated groups, whereas the over-expression in AKBA- was significantly less than that of in ABA-, and BA-treated MCF-10A cells. In summary, the ABA represented superior apoptotic activity compared to the AKBA, since it did not cause apoptosis in non-tumorigenic non-transformed breast epithelial cell line among over-mentioned pathways.

### Relative protein expression

**Figure 5:**
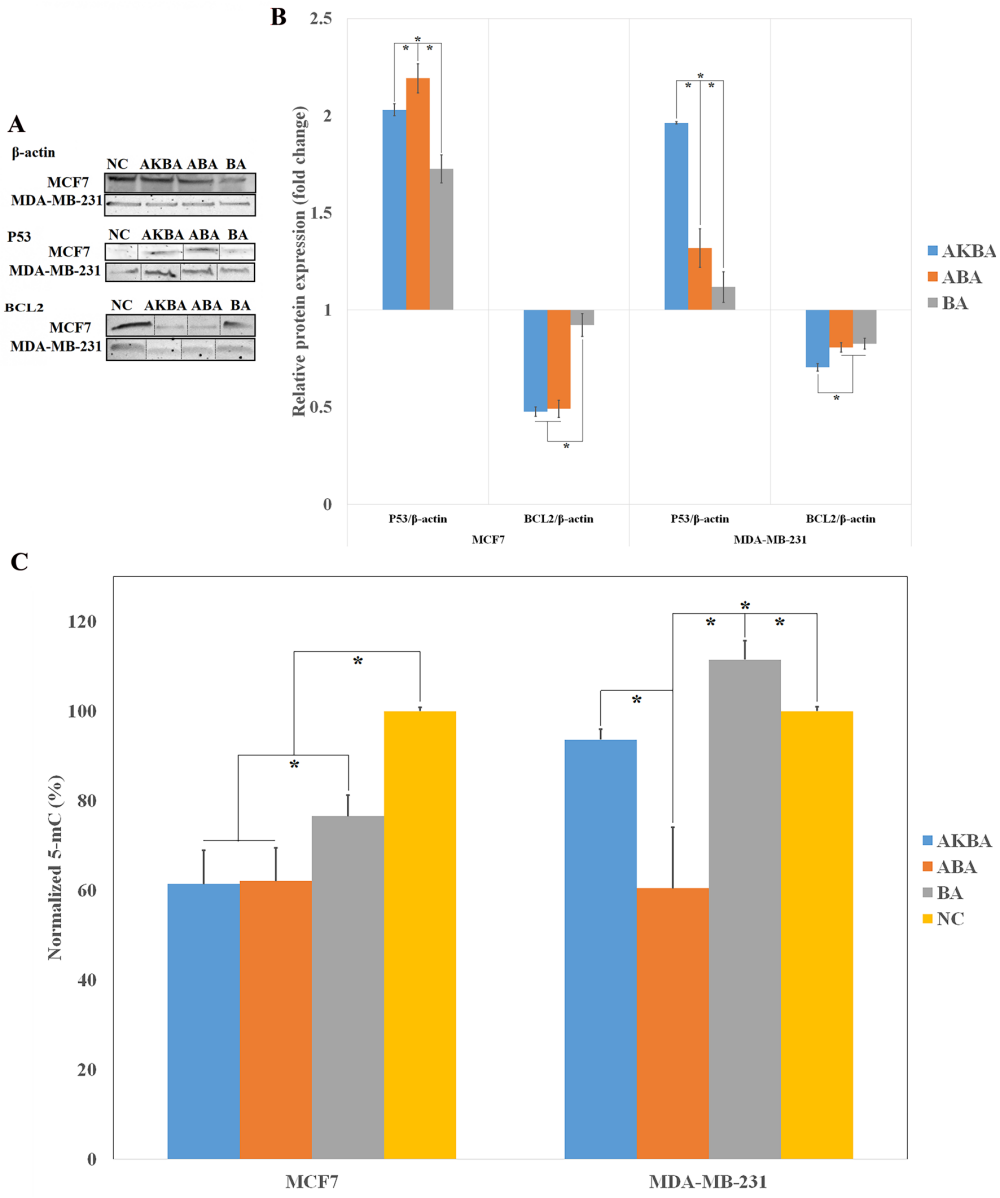
(**A**) Representative western blot images, showing the protein expression of *P53* and *BCL2* in treated and non-treated MCF-7, and MDA-MB-231 cells. Dashed lines represent cropped images. Uncropped images are presented in Supplementary Fig. S1. (**B**) Relative protein expression obtained by quantitative analysis of western blot assay (N value = 3). *indicates a significant difference (P-value ≤ 0.05). (**C**) The Global DNA methylation assay of BA-, ABA-, and AKBA-treated MCF-7, and MDA-MB-231 cells compared with the negative control group. *indicates a significant difference (P-value ≤ 0.05).

As shown in Fig. 5A,B, both ABA- and AKBA-treated MCF-7 cells over-expressed P53 protein. The maximal expression of P53 protein was detected in ABA-treated MCF-7 cells. Moreover, both ABA and AKBA could significantly suppress the *BCL2* protein in MCF-7 cells in comparison with the NC. These are in a complete agreement with the gene expression results. With regard to MDA-MB-231 cells, while the *P53* was up-regulated 1.96 times in AKBA group, the ABA could just induce 1.32 times over-expression of *P53* protein. Also, a significant drop in BCL2 protein was detected in AKBA-treated MDA-MB-231 cells.

### Global DNA methylation

The normalized 5-mC percentages were depicted in Fig. 5C. Whereas, the normalized 5-mC percentages in BA-, ABA-, and AKBA-treated MCF-7 cells was significantly less than that of in NC, the maximal decrement in 5-mC were seen in ABA- and AKBA-treated MCF-7 cells. On the other hand, the ABA-induced global DNA methylation in MDA-MB-231 cells was remarkably less than AKBA. Also, an increment in 5-mC percentage of BA-treated MDA-MB-231 cells was observed in comparison to the NC.

## Discussion

Our toxicity results are in parallel with those reported by Schmiech et al., indicating that AKBA and ABA exhibited the highest cytotoxic efficacy against MDA-MB-231 cells, in vitro^30^. Moreover, BA has represented the minimal anti-proliferative activity against MDA-MB-231 cells with 69% of live cells. AKBA has shown the maximal toxicity of MCF-10A cells with 45% dead cells. Besides, ABA significantly revealed less toxicity in comparison with the AKBA. Kumar et al. have demonstrated that ABA could be considered as a cytostatic rather than a cytotoxic drug and can be applied in chemo-preventive intervention approaches^20^. Furthermore, DAPI staining also confirmed that ABA, and AKBA could be effective in the inhibition of proliferation and promotion of apoptosis in both cancerous cell lines. Regarding MCF-10A and MDA-MB-231 cells, ABA- and AKBA- treated cells look like smaller than other groups (Fig. 1B,D), which is possibly due to the cells shrinkage and pyknosis happen during the early stage of apoptosis^31^. On the other hand, higher proliferation rate and increment in the DNA content are responsible for the larger size of the cells treated with BA, as there is a strong correlation between the genome size and the cell size^32^.

Cell cycle assay result is in parallel with the reported data by Kunnumakkara et al. which indicated that AKBA accumulated cells in Sub-G1 phase^33^. Also, both ABA and BA increased the Sub-G1 phase in MDA-MB-231 cells, while there were no notable difference between them. Syrovets et al. have indicated that ABA and AKBA induced the sub-G0/G1 phase in prostate cancer cells^34^.

The AKBA apoptosis assay results confirming the reported finding by Li et al.^35^. In addition, the early- and late-apoptotic induction of ovarian and colorectal cancer cells using AKBA have been proven by Lu et al. and Toden et al., correspondingly^21,36^. Taking together, AKBA compared to the ABA represented superior apoptosis activity, while it caused both early- and late-apoptosis.

The preventive effect of AKBA on cells migration is in agreement with previous literatures^21^. According to the presented results, BA was the only compound which caused no inhibition in the migration of MCF-10A cells, while it prevented cancer cells migration. This may be considered beneficial since it could aid the healing rate of post-surgical wound. Moreover, the inhibition in the migration of ABA-treated MDA-MB-231 cells was significantly more than that of BA-treated cells.

It seems that *P53* pathway is responsible for the cytotoxicity induced by AKBA or ABA. Additionally, the apoptosis mediated by P53 proves the anti-carcinogenic impact of ABA and AKBA as a potential therapeutic agent for breast cancer. Although AKBA could greatly induce apoptosis in cancer cells (MCF-7 and MDA-MB-231), it was toxic for MCF-10A cells. In agreement with our results, Jiang et al. have reported that AKBA induced apoptosis in both cancer and non-tumorigenic non-transformed breast epithelial cell lines^37^. Liu et al. revealed the over-expression of *P21* and *P53* in colon cancer cells after treatment with AKBA^38^. Our data indicated that acetylated compounds (AKBA and ABA) were more effective against cancer cells compare with BA which confirmed the Schmiech et al.'s finding^30^.

Several mechanisms underlying MDR have been identified such as DNA modification. Mutation in P53 gene may disturb pro-apoptotic balance resulting in MDR^39^. This correlation has been demonstrated among 60 cell lines and more than 100 anti-cancer drugs^40^. Thus, according to the results both AKBA and ABA are potent to be utilized as natural chemo-sensitizers to reverse the MDR via activating P53 pathway. Moreover, researchers provided an evidence showing that AKBA may overcome ovarian cancer resistance^21^.

The apoptosis resulted by P53 would happen through a mitochondrial pathway that is mainly mediated by BCL2 proteins, such as BAX. BAX and BCL2 as pro-apoptotic and anti-apoptotic markers, activates undelaying mechanism responsible for apoptosis via creating a heterodimer^41^. The ratio of Bax/BCL2 expression level is assumed as an indicator of the susceptibility to apoptosis^42^. The ratio of Bax to BCL2 in AKBA- and ABA-treated MCF-7 cells were obtained 2.12 ± 0.15, and 1.77 ± 0.13, respectively, which were significantly higher than that of NC. Similarly, In case of MDA-MB-231 cells, the Bax/BCL2 ratio in AKBA- and ABA- treated was significantly greater than that of NC (1.82 ± 0.06, and 1.38 ± 0.07, respectively). The increased ratio of Bax/BCL2 expression levels in AKBA compared to the ABA group suggested superior apoptosis robust in AKBA group for both cancerous cell lines.

The results indicated that global DNA methylation in MCF-7 cells level was affected by AKBA and ABA, while in MDA-MB-231 cells it was altered just by ABA. Alterations or variations in global DNA methylation are considered as important criteria in cancer epigenomics because they are typically associated with cell survival, proliferation, differentiation, and cancer progression^43^. Global hypomethylation and promoter hypermethylation in tumor suppressor genes have been detected in most cancers^44^. The functional effects of ABA in the inhibition of global DNA methylation in both cancerous cells make it a potential epigenetic operator for prospective clinical means in mammary carcinoma breast cancer therapy.

A common epigenetic alteration which happens in most types of cancers is DNA Hyper-methylation in CpG Islands of the promoter regions in tumor suppressor genes. Despite genomic alterations, abnormal methylation of methylation-silenced genes is considered reversible using therapeutic agents^45^. DNA methyltransferase inhibitor, 5-aza-2′-deoxycytidine, has been broadly applied to treat human malignancies via reversing DNA hyper-methylation^45^. As epigenetic alterations would occur at the earliest carcinoma stages, there is a rising tendency to employ demethylation inducers as cancer preventing agents rather than therapeutic cues^45,46^. Accordingly, ABA which could cause demethylation in both types of cancerous cells, is potent to be applied for cancer prevention with minimal adverse side effects.

Corresponding to the study presented by Du et al. ABA revealed much greater in vivo bioavailability compared to the AKBA^47^. Due to the superior intrinsic properties shown by either AKBA or ABA, it sounds like that the combination of AKBA and ABA may improve the pharmacodynamics properties of boswellic acid derivatives. To our knowledge, this is the first comprehensive study that compares the apoptotic, toxicity, anti-metastatic, and epigenetic modulatory effects of boswellic acid derivatives. In this study, ABA and AKBA revealed complementary properties which their synergism is expected to be beneficial in breast cancer prevention and therapy. While ABA revealed a more epigenetic-modulatory and less toxicity against MCF-10A, AKBA represented more cytotoxic, apoptotic, and anti-metastatic activity against breast cancer cell lines.

## Materials and methods

### Extraction and isolation of AKBA, β-ABA, and β-BA

The *Boswellia sacra Flueck* resins samples (1.4 kg) were collected from the various locations in Dhofar, Oman. The specimen (BSHR-01, April 2020) was deposited in the herbarium of the Natural and Medical Sciences Research Center (NMSRC), University of Nizwa, Oman.

The air-dried powder resin of *B. sacra* (1.4 kg) was extracted with methanol (MeOH, 2.0 L) at room temperature (three times) and evaporated under reduced pressure to yield a yellow semi-solid residue (900 g). The crude MeOH extract was successively fractionated into n-hexane (472.5 g), ethyl acetate (EtOAc, 388.0 g) and aqueous (35.0 g). The EtOAc fraction was subjected to column chromatography (CC) using isocratic mobile phase viz., 10%, 20%, 30%, 40% and 50% EtOAc/n-hexane to get eighteen fractions (BSF_1–18_). After taking TLC, four sub fractions (BSF_4-8_) were combined and further chromatographed on CC to afford a mixture of two compounds **ABA** (220 mg) and **BA** (160 mg) using 20% and 30% AcOEt/n-hexane system as a mobile phase along with some semi-pure compounds, which were later purified through preparative high performance liquid chromatography (prep. HPLC) using CHCl_3_ solvent. Sub fractions (BSF_10–16_) were combined due to their similar TLC profile and further subjected on CC using EtOAc/n-hexane with increasing polarity (30, 35, 40, 45 and 50%) to afford compound **AKBA** with some impurities which was later purified through prep. HPLC using CHCl_3_ solvent. The structure elucidation of all these compounds was achieved by extensive spectroscopic techniques including 1D (^1^H and ^13^C) and 2D (NOESY, COSY, HMBC and HSQC) NMR and mass spectrometry and comparison of the spectral data of known compounds with those reported in literature^48,49^.
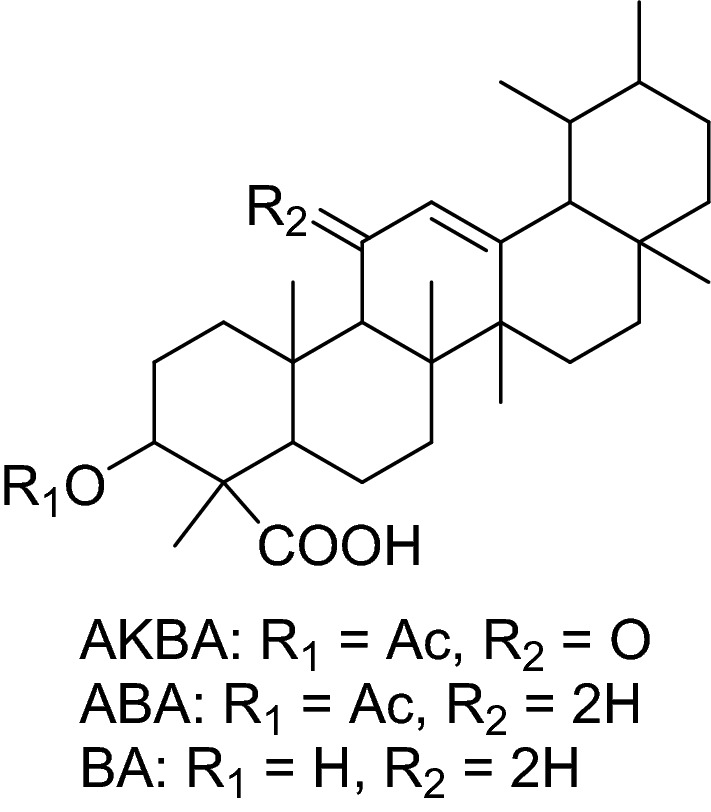


### Cell culture

MDA-MB-231 and MCF-7 cell lines were acquired from the American Type Culture Collection (ATCC) and MCF-10A was purchased from Iranian Biological Resource Center (IBRC) (Tehran, Iran). MDA-MB-231was cultured in Dulbecco's modified Eagle's medium (DMEM)/F12 (1:1) enriched with 100 U/ml penicillin and 100 μg/ml Streptomycin, 10% fetal bovine serum (FBS; Gibco), and L-glutamine (6 mM). MCF7 was cultivated in minimal essential medium (MEM) containing 100 U/ml penicillin and 100 μg/ml Streptomycin, 10% fetal bovine serum (FBS; Gibco). MCF-10A was maintained in DMEM/F12 (1:1) containing 5% horse serum, 20 ng/mL EGF, 10 μg/mL insulin, 50 μg/mL hydrocortisone and 100 U/ml penicillin and 100 μg/ml Streptomycin. All cells were incubated at 37 °C in a humidified atmosphere containing 5% CO_2_.

### Cell proliferation assays

Each cell line was seeded in 96-well plates at 10^4^ cells. Once adhered, cells were treated with AKBA, ABA, BA, and DMSO as a negative control, with the various dosages. After 24 h of treatment, cell viability was evaluated using a 3-(4,5-dimethylthiazole-2-yl)-2,5-diphenyl tetrazolium bromide (MTT) assay kit (Bio-idea, Tehran, Iran). The optical value was read by using of a BIO-RAD (Hercules, CA, USA) reader at a wavelength of 570 nm.

After 24 h of cells treatment with 65 μM AKBA, ABA, and BA, the viability was further assessed utilizing the live and dead assay kit (ThermoFisher Scientific, USA) according to the manufacturer's protocol. Using this assay, live cells were distinguished from the dead cells by fluorescence microscopy (Optika XDS 3FL4, Italy) with excitation and emission of green (ex/em 494/530 nm for Calcein AM) for live cells and red (ex/em 528/645 nm for EthD-1) fluorescence for dead cells^50^. For nuclear staining, the 24 h-treated cells were stained with 4′,6-diamidino-2-phenylindole (DAPI) dye (Sigma-Aldrich, USA), following of rinsing the cells with PBS. Finally the cells were evaluated using a fluorescent microscope (Optika XDS 3FL4).

### Cell cycle assay

Cells were cultivated in 12-well plate at 2 × 10^5^ cells/well and following 24 h of seeding, they were treated with 65 μM AKBA, ABA, and BA for an additional 24 h. Cells were collected, fixed in 70% ethanol at 4 °C overnight, and then incubated with Guava Cell Cycle Reagent (Cat. No.4500-0220) for 30 min at room temperature. Cell cycle analysis was achieved utilizing a flow cytometer (guava easyCyte™ HT System, Millipore, Bedford, MA, USA)^51^.

### Apoptosis assay

Cell apoptosis was evaluated 24 h after treatment with 65 μM AKBA, ABA, and BA. Treated cells were raised by trypsinization, washed in ice-cold PBS. Subsequently, cells were re-suspended in the 1X-binding buffer to a concentration of 1.25 × 10^5^ cells. eBioscience™ Annexin V-FITC apoptosis detection kit (Thermo Fisher Scientific) was employed to investigate cell apoptosis according to the manufacturer's protocol. Briefly, cells were stained with AnnexinV-FITC and incubated at room temperature in dark condition for 15 min. The cells were then rinsed and stained with propidium iodide (PI) followed by apoptosis assessment using a flow cytometer (guava easyCyte™ HT System).

### In vitro migration assays

#### Scratch assay

Cell migration was determined by making identical scratch areas for all three-cell lines. Cells were cultivated to 80% confluence in 12-well plates, then an artificial scratch was made by 10 µl sterile pipette tips. Afterward, cells were washed twice with PBS to remove detached cells. The culture medium was replaced by media containing 65 μM of AKBA, ABA or BA for 24 h. Following changing the media with the original medium, migration to the scratched area was imaged at 12, 24, 36, and 48 h after wounding. Finally, the closure rate in the treated cells was compared with the negative control group, cells treated with DMSO and the migration inhibition was calculated by ImageJ software.

#### Transwell migration assay

MDA-MB 231 cells were seeded at a frequency of 2.7 × 10^5^ cells onto the topside of 8 µm-pore transwell membrane in 24-well tissue culture plates. Following 24 h culture, the media was changed to a serum-free DMEM medium supplemented either with AKBA, ABA or BA (65 µm). After 24 h, the media was substituted with a serum-free DMEM medium without compounds. MDA-MB-231 cells were tagged with a Cell Tracker Red CMTPX Dye (Thermo Fisher Scientific) according to the producer’s protocol. The MDA-MB-231 cells migration to the lower side of the cell insert membrane were followed utilizing a fluorescence microscope (Optika XDS 3FL4). The quantity of migrated cells was obtained using ImageJ 1.52a software (NIH, Bethesda, MD, USA). Afterward, the cell insert membrane were fixed and dehydrated with 2.5% glutaraldehyde in PBS and increasing concentrations of ethanol, respectively. The photos from the cells that migrated to the lower side of the inserts were taken using an electron scanning microscope (SEM, JEOL Ltd., Tokyo, Japan)^50^.

### RNA isolation and real‑time PCR

After 24 h of MCF-7, MDA-MB-231 and MCF-10A cells treatment in 6-wells plate, the mRNAs were obtained using RNX- Plus Solution (Sinaclon, Iran). Afterward, the cDNA was synthesized using High-Capacity cDNA Reverse Transcription Kit with RNase Inhibitor, according to the manufacturer's instructions (ThermoFisher Scientific). The expression level of *P53*, *P21*, *BAX* and *BCL2* were studied by Real-time PCR in comparison with the negative control. PCR amplification was implemented using SYBR™ Green PCR Master Mix (ThermoFisher Scientific) and Beta-actin transcripts served as endogenous controls. Changes in mRNA expressions were normalized to the relevant internal control and finally measured utilizing the 2^-ΔΔCt^ methodology. The QuantStudio (applied biosystems by ThermoFisher Scientific) was used for quantitative mRNA transcript expressions. Primers used for qPCR reactions are detailed in Table 1.

**Table 1:** The sequence of Beta-actin, *P53*, *P21*, *BAX*, and *BCL2* primers.

Name	Sequence forward	Sequence reverse
Beta-actin	CTTCCTTCCTGGGCATG	GTCTTTGCGGATGTCCAC
P53	GGAGTATTTGGATGACAGAAAC	GATTACCACTGGAGTCTTC
P21	CCAGCATGACAGATTTCTACC	AGACACACAAACTGAGACTAAGG
BAX	CAAACTGGTGCTCAAGGC	CACAAAGATGGTCACGGTC
BCL2	GTACTTAAAAAATACAACATCACAG	CTTGATTCTGGTGTTTCCC

### Western blot

The MCF-7 and MDA-MB-231 cells following 24 h treatment with AKBA, ABA or BA were trypsinized and lysed with RIPA buffer and protease inhibitors (ThermoFisher Scientific). The protein concentrations were checked by Pierce™ Rapid Gold BCA Protein Assay Kit (ThermoFisher Scientific). The lysates were incorporated with Laemmli sample buffer. Western blot analysis of *P53* and *BCL2* was achieved by loading 50 μg of total proteins and were run by 10% acrylamide gel (Bolt Bis–Tris Plus gels) (Invitrogen, USA) at a constant voltage of 200 V. Following SDS-PAGE, the proteins from the gel were transferred to Nitrocellulose membranes by Pierce power blotter (ThermoFisher Scientific) with a high M.W. pre-programmed approach. The membranes were later blocked with BSA dissolved in TBS/Tween-20 (5% BSA, 0.5% Tween-20 for 1 h), accompanied by immunoblotting with *P53* monoclonal antibody (1/1500 dilution) and *BCL2* monoclonal antibody (1/50 dilution) (ThermoFisher Scientific) as well as beta actin monoclonal antibody as a loading control (ThermoFisher Scientific) for overnight. Afterward, blotting was followed by incubation with goat anti-mouse IgG horseradish peroxidase-conjugated secondary antibody (ThermoFisher Scientific, 1/5000 dilution). The bands were detected using enhanced chemiluminescence (Abcam, USA) with the iBright™ 1500 Imaging System (Invitrogen)^52^.

### Global DNA methylation assay

MCF-7 and MD-MB-231 cells were treated with 65 μM AKBA, ABA, or BA for 24 h and later compiled for genomic DNA isolation using the GeneJET Genomic DNA Purification Kit (Thermo Fisher Scientific). The Global DNA methylation levels were obtained utilizing the MethylFlash Global DNA Methylation Quantification Kit (Epigentek, USA) according to the manufacturer’s instructions.

### Statistical analysis

All experiments were performed in triplicate. Data have been presented as average ± standard deviation. The results were analyzed using one-way analysis of variance and Tukey’s post-hoc test. The comparison investigations were performed using Minitab 17 software (Minitab, Pennsylvania, USA). The differences were considered statistically significant at P < 0.05.

### Ethics declarations

According to international and local guidelines^53^, the resin samples were collected with care and trees were treated ethically. During the harvesting, the local environment was not harmed. Permission was granted by Ministry of Environment, Muscat, Sultanate of Oman (6210/10/73, 19th March 2019) to collect biological materials for science purpose. The current study did not involve endangered or protected species. The plant was identified by Mr. Mohammed Al-Broumi, botanist at the Natural and Medical Sciences Research Center, University of Nizwa, Oman, and voucher specimen (BSHR-01, April 2020) was deposited in the herbarium of the center.

## Conclusion

According to this study, AKBA revealed strongest in vitro anti-proliferative, anti-metastatic and apoptotic activity against cancer cells. Despite this, ABA showed the safest properties since it did not induce apoptosis in MCF-10A cells. Additionally, ABA could lessen the global DNA methylation in both MCF-7 and MDA-MB-231 cells, which is favorable for cancer chemoprevention. It is concluded that the synergism of ABA as a potential epigenetic operator and AKBA as an apoptotic factor may be beneficial in breast cancer prevention and therapeutic strategies with less MDR and greater selectivity compared to conventional chemotherapeutic approaches. Further study warranted studying the synergistic effect of ABA, and AKBA to achieving a potential drug for breast cancer therapy.

## Supplementary Information

Supplementary Information.

## Data Availability

The data that support the findings of this study are available from the corresponding author, [Ahmed Al-Harrasi], upon reasonable request.
