# Supplementary material for: Comparative study of the cytotoxicity, apoptotic, and epigenetic effects of Boswellic acid derivatives on breast cancer
Source: Sci Rep. 2022 Nov 21;12:19979. doi: 10.1038/s41598-022-24229-y (PMC9678894; doi:10.1038/s41598-022-24229-y)
Supplement: Supplementary file 1 — Supplementary Information. [file 41598_2022_24229_MOESM1_ESM.docx]

Title:

**Comparative study of the cytotoxicity, apoptotic, and epigenetic effects of Boswellic Acid derivatives on breast cancer**

*Fatemeh Jamshidi-adegani*^a^*, Shokoofeh Ghaemi*^a, b^*, Sulaiman Al-Hashmi*^a^*, Saeid Vakilian^a^, Juhaina Al-kindi*^a^*, Najeeb Ur Rehman*^c^*, Khurshid Alam*^d^*, Khamis Al-Riyami*^c^*, Rene Csuk^e^, Ehsan Arefian*^b^**,  Ahmed Al-Harrasi^c^***

^a^Laboratory for Stem Cell & Regenerative Medicine, Natural and Medical Sciences Research Center, University of Nizwa, Nizwa, P. O. Box: 33, PC 616, Oman

^b^ Department of Microbiology, School of Biology, College of Science, University of Tehran, Tehran, Iran

^c^Natural products Laboratory, Natural and Medical Sciences Research Center, University of Nizwa, Nizwa, P. O. Box: 33, PC 616, Oman

^d^Department of Mechanical and Industrial Engineering, Sultan Qaboos University, 123, Muscat, Oman

^e^Organic Chemistry, Martin-Luther-University Halle-Wittenberg, Kurt-Mothes-Str. 2, D-06120 Halle (Saale), Germany


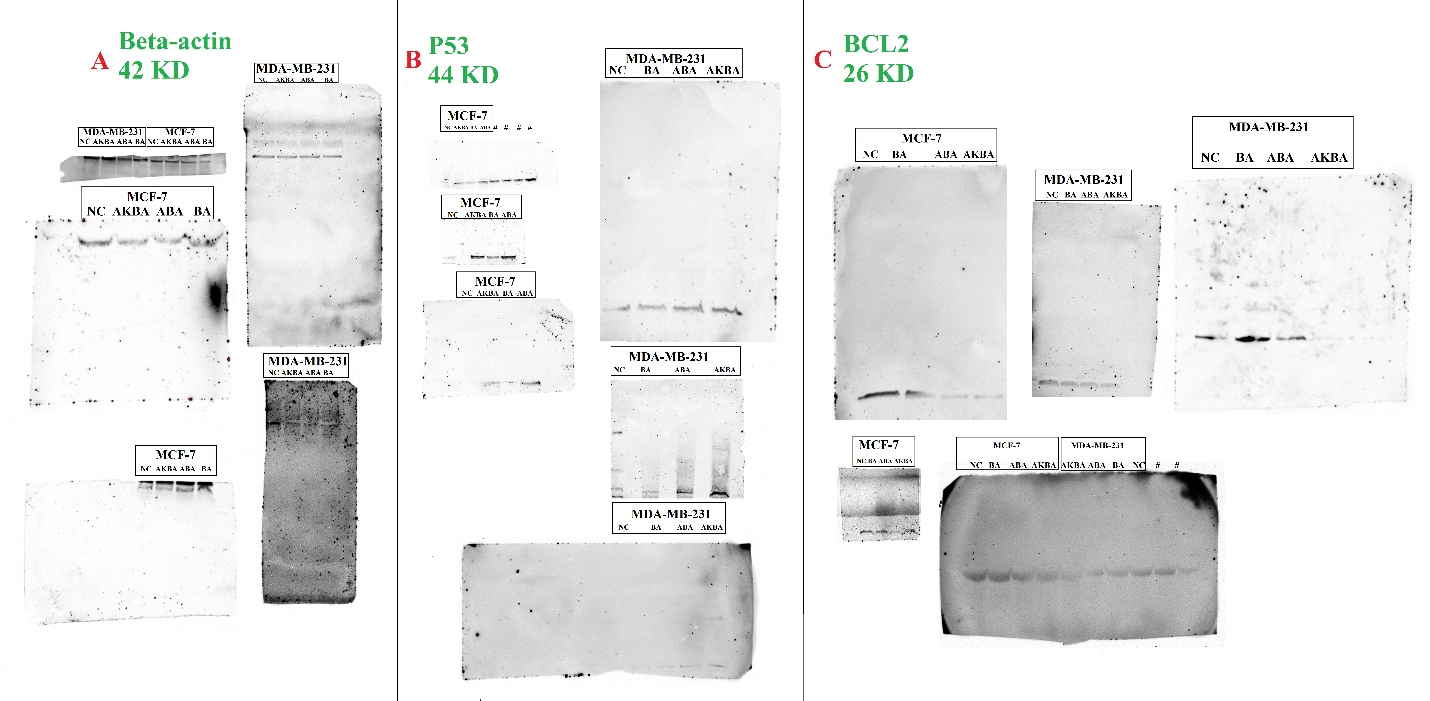


Figure S1. Uncropped images of western blotting for Beta-actin (A), P53 (B), and BCL2 (C) with three replicates. **#** indicates groups that are not belonged to this study, but they were run in the same blot. Some membranes were cut prior to hybridization with the primary antibody.
